# IL-21 Induces an Imbalance of Th17/Treg Cells in Moderate-to-Severe Plaque Psoriasis Patients

**DOI:** 10.3389/fimmu.2019.01865

**Published:** 2019-08-07

**Authors:** Yuling Shi, Zeyu Chen, Zihan Zhao, Yingyuan Yu, Huayu Fan, Xiaoguang Xu, Xiaolin Bu, Jun Gu

**Affiliations:** ^1^Department of Dermatology, Shanghai Tenth People's Hospital, Tongji University School of Medicine, Shanghai, China; ^2^Institute of Psoriasis, Tongji University School of Medicine, Shanghai, China; ^3^Department of Dermatology, Shanghai Changhai Hospital, Second Military Medical University, Shanghai, China; ^4^Department of Dermatology, Gongli Hospital, Second Military Medical University, Shanghai, China

**Keywords:** psoriasis, Th17 cells, Treg cells, IL-17A, IL-21, IL-22

## Abstract

**Background:** Psoriasis is a chronic immune-mediated inflammatory skin disease, with over-activated interleukin (IL)-17-producing CD4^+^ T cells (Th17) and repressed regulatory T (Treg) cells. IL-21 is a Th17-related cytokine and plays an important role in the pathogenesis of psoriasis. However, the mechanism by which IL-21 affects the pathogenic progress of psoriasis remains poorly understood.

**Methods:** IL-21 and IL-21 receptor (IL-21R) expression in normal and psoriatic lesional skin were determined by immumohistochemical staining, immunofluorescence staining, and western blotting. The levels of IL-21, IL-17A, and IL-22 in the culture supernatants were measured by enzyme-linked immunosorbent assay (ELISA). The level of IL-10 in the culture supernatants was measured by cytometric bead array (CBA). The mRNA expression levels were assessed by quantitative polymerase chain reaction (qPCR). CD4^+^ T cells were isolated from the peripheral blood mononuclear cells (PBMCs) from the psoriasis patients and healthy individuals and then treated with or without IL-21 for 3 days. The proportions of Th17 and Treg cells were determined by flow cytometric analysis.

**Results:** IL-21 and IL-21R were highly expressed in the lesional skin and peripheral blood of psoriasis patients. IL-21 promoted CD4^+^ T cells proliferation and Th17 cells differentiation and inhibiting Treg cells differentiation by upregulating RORγt expression and downregulating Foxp3 expression, with increased expression and secretion of IL-17A and IL-22. The proportion of Treg cells was negatively correlated with that of Th17 cells in psoriasis patients.

**Conclusion:** Our results suggest that IL-21 may promote psoriatic inflammation by inducing imbalance in Th17 and Treg cell populations.

## Introduction

Psoriasis is a chronic inflammatory skin disease that affects ~2 and 0.47% of the population in Europe and North America (1) and China (2), respectively. Because of genetic and environmental factors, the communication between keratinocytes and immune cells, including T cells, dendritic cells, macrophages, and neutrophils, is impaired in psoriasis (3). The accumulation of immune cells in the psoriatic lesional skin, such as Th17 cells, can produce abundant inflammatory mediators, such as IL-17A, that lead to the accumulation of neutrophils and impaired differentiation and hyperproliferation of keratinocytes, resulting in epidermal hyperplasia (3). The IL-23-IL-17A axis plays a critical role in the pathogenesis of psoriasis, which is demonstrated by the substantial success of biological agents targeting IL-23 and IL-17A in the treatment of psoriasis (1, 4–9). The pro-inflammatory cytokine IL-17A stimulates keratinocytes to proliferate and secrete abundant inflammatory cytokines and chemokines, leading to the hyperproliferation of keratinocytes and the further recruitment of neutrophils, Th17 cells, dendritic cells, and other inflammatory cells (10–13). The further recruitment of immune cells leads to an aggravating circle of chronic skin inflammation (3).

The main source of IL-17A in psoriasis patients is Th17 cells, and these significantly accumulate in the lesional skin and clinically resolved psoriatic lesions of psoriasis patients (14). Th17 cells can secrete other pro-inflammatory cytokines, such as IL-22, to promote the development of psoriasis (1). Treg cells are a subset of T cells that suppress the inflammation induced by other T cells, such as Th17, in autoimmune diseases (15) and impaired Treg cells that are found in psoriasis patients (16–18). Foxp3 is essential for the development, maintenance, and function of Treg cells and inactivating mutations in Foxp3 lead to the spontaneous autoimmunity with a scurfy phenotype in mice and immune dysregulation, polyendocrinopathy, enteropathy, X-linked (IPEX) syndrome in humans (19). The conditional deletion of a Foxp3 allele in mature Treg cells leads to effector T cells that can cause inflammation (19). Furthermore, the conversion of Treg cells into Th17 cells has been reported in a mouse model of psoriasis (20).

IL-21, a Th17-related cytokine, which belongs to the type I four-α-helical-bundle family of cytokines, is reported to promote Th17 differentiation, enhances the function of Th17 cells, and inhibits the generation of Treg cells (21–24). The signals of IL-21 is via a receptor composed of IL-21R and the common cytokine receptor γ-chain, γ_c_ (25). IL-21 is primarily produced by CD4^+^ T cells, such as Th17 and follicular helper T cells, and natural killer T cells (25). IL-21 has multiple actions on adaptive and innate immune cells, including T, B, NK, NKT, and dendritic cells (25). Although IL-21 has been widely studied in other autoimmune diseases, the role of IL-21 is still unclear in psoriasis. Thus, in the present study, we investigated the effects of IL-21 on CD4^+^ T cells of psoriasis patients.

## Materials and Methods

### Patients

This study was performed at the Shanghai Tenth People's Hospital and Shanghai Changhai Hospital and was approved by the ethics committees of those institutions (IRB approval number: SHSY-IEC-3.0/15-67/01). The psoriatic skin samples were obtained by excisional biopsy from patients with moderate-to-severe plaque psoriasis who were diagnosed based on the clinical and histopathological criteria. The disease activity of the patients was assessed using a psoriasis area and severity index (PASI) score at the time of blood collection and categorized as moderate (11–20) or severe (>20). There were no other autoimmune diseases, systemic diseases, or active infections in any of the participants. All the patients were not treated on systemic therapy for at least 4 weeks and on topical therapy for at least 2 weeks. All the participants were enrolled in accordance to the Declaration of Helsinki principles for research involving human subjects. Consent was obtained from all the participants and clinical information, blood samples, and skin biopsies were collected.

### Isolation of Peripheral Blood Mononuclear Cells (PBMCs)

Lymphocyte separation medium (PAA Laboratories GmbH, Austria) was used for the isolation of PBMCs from the blood samples of psoriasis patients and healthy individuals according to the manufacturer's instruction. The survival rate of PBMCs was determined using Trypan Blue (Sangon Biotech, Shanghai, China). After isolation, PBMCs were used for detecting the mRNA expression of IL-21, IL-21R, other cytokines, and transcriptional factors, determining the ability of cytokines production, determining the proportion of T cell subpopulation, and purifying CD4^+^ T cells for other experiments.

### Enzyme-Linked Immunosorbent Assay (ELISA)

Levels of IL-17A and IL-22 in the supernatant of cultured CD4^+^ T cells were measured using a human IL-17A ELISA kit (R&D Systems, Minnesota, USA) and human IL-22 ELISA kit (R&D Systems, Minnesota, USA) according to the manufacturer's instructions.

### Cytometric Beads Array for Detecting Cytokines Levels

Level of IL-10 in the supernatant of cultured CD4^+^ T cells was determined suing Cytometric Beads Array (BD Pharmingen) according to the manufacturer's instructions. Data were acquired on FACS Canto-II (BD Bioscience, USA). Data were analyzed using FCAP Array software (BD Bioscience).

### Flow Cytometric Analysis and Intracellular Staining

Skin single cell suspension was prepared as previously described (11). Anti-CD4-Fitc, anti-CD3-PE-cy7, anti-CD16-APC, anti-CD56-APC, anti-IL-17A-PE, anti-IL-21-Alexa Fluor^®^ 647, anti-IL-21-PE, anti-IL-10-PE-cy7, anti-TGF-β-PE, anti-CD25-APC, anti-CD25-PE-cy7, anti-CD25 PE, anti-Foxp3-APC, and anti-Foxp3-PE antibodies were purchased from eBioscience. Anti-IL-21R-PE antibody was purchased from BD Biosciences (USA). Anti-CD19-BV605 antibody was purchased from Biolegend (USA). Isotype antibodies were used as the control. For intracellular staining, the cells were cultured in R10 medium (RPMI1640 with 10% fetal bovine serum, 100 U/ml penicillin, 100 mg/ml streptomycin, and 2 mM glutamine) in the presence of 50 ng/ml phorbol myristate acetate (PMA, Sigma, USA), 1 μg/ml ionomycin (Sigma, USA) for 2 h; then 0.7 μl/mL Golgistop (BD Biosciences, USA) was added and the cells were incubated for an additional 3 h. For surface staining, the cells were washed with staining buffer (PBS containing 2% fetal bovine serum) and stained with fluorescence-conjugated antibodies against surface antigens at room temperature for 15 min. Thereafter, the cells were washed with staining buffer for two times, fixed with fixation buffer at room temperature for 20 min, permeabilized, and stained with fluorescence-conjugated antibodies against intracellular antigens at room temperature for 20 min. For detecting Foxp3 expression, fixation/permeabilization diluent and concentrate (eBioscience) were used. The data were acquired on FACS Calibur (BD Biosciences) or FACS Canto-II and analyzed using CELLQuest software or Flow Jo software (Tree Star).

### Western Blotting

Strong radio immunoprecipitation (RIPA) lysis buffer (PIERCE) was used for the lysing of tissue samples from psoriasis patients and healthy individuals. Whole tissue lysates were subjected to 10% SDS-polyacrylamide gel electrophoresis and transferred onto polyvinylidene difluoride membranes. Then, the membranes were incubated with the indicated antibodies. Primary antibody against IL-21 was purchased from Novus Biologicals, and IL-21R and β-actin were purchased from Abcam. The secondary antibodies conjugated with HRP were used at 1:1,000 dilutions (ZSGB-BIO, China). The protein bands were detected with the Odyssey Infrared Imaging System (LI-COR, USA).

### Immunohistochemical Analysis of Human Skin Sections

Skin tissues from psoriasis patients and healthy individuals were fixed in 4% paraformaldehyde at room temperature, embedded in paraffin, and sectioned at a thickness of 5 μm. After paraffin depletion and rehydration, the sections were washed with distilled water. For antigen retrieval, the sections were autoclaved at 120°C for 15 min in 1 mM EDTA buffer (pH 8.0) and cooled at room temperature for 20 min. After antigen retrieval, the sections were washed with PBS for three times. For the inactivation of intrinsic peroxidases, the sections were incubated in 3% H_2_O_2_ in distilled water at room temperature for 10 min. Then, the sections were washed with PBS for three times, incubated in 5% goat serum at room temperature for 10 min, and stained with primary antibodies: rabbit anti-human IL-21 (Novus Biologicals), rabbit anti-human IL-21R (Abcam, MA, USA), or rabbit anti-human IL-17A (Abcam, MA, USA). The sections were stained with the antibodies at 4°C overnight. After washing with PBS for three times, the sections were incubated with polymer helper at room temperature for 20 min. After washing with PBS for three times, the sections were stained with poly peroxidase-anti-rabbit IgG (Abcam, MA, USA) at room temperature for 20 min. After washing with PBS, the secondary antibody was further visualized with fresh DAB buffer, and the sections were counterstained with hematoxylin.

### Immunofluorescence Staining

The skin sections were prepared as described in the immumohistochemical analysis of human skin sections. After incubating with goat serum, the sections were incubated with PBS for 10 min and stained with rabbit anti-human IL-17A (Abcam, MA, USA) or rabbit anti-human IL-21 (Novus Biologicals, Colorado, USA) at 4°C overnight. After washing with PBS for three times, the sections were incubated with mouse anti-human CD4 (Abcam, MA, USA) at 4°C overnight. After washing with PBS for three times, the sections were incubated with secondary antibodies [FITC-conjugated goat polyclonal to mouse IgG (Abcam, MA, USA) or Cy5-conjugated goat polyclonal to rabbit IgG (Abcam, MA, USA)] at 37°C for 1 h protecting from light. The nuclei were stained with DAPI, and the images were acquired with a fluorescent microscope (Leica).

### RNA Extraction and Real-Time Quantitative PCR (qPCR)

Total RNAs were extracted from the frozen tissues and PBMCs using TRIzol solution (Invitrogen, CA, USA) and reverse-transcribed with PrimeScript RT Reagent Kit (TaKaRa Biotechnology, Dalian, China). The RNA expression levels were detected by SYBR Green qPCR analysis (KAPA, USA). The primer sequences were as follows: β-actin (FW 5′-TGG CAC CCA GCA CAA TGA A-3′, RV 5′-TAA GTC ATA GTC CGC CTA GAA GCA-3′), IL-21 (FW 5′-CCA AGG TCA AGA TCG CCA CA-3′, RV 5′-TTC TGG AGC TGG CAG AAA TTC A-3′), IL-21R (FW 5′-AGA CCC TCA ATA AAC GTC AGC TTC C-3′, RV 5′-TCG CTG ACG ATT GAT GTT CTC AC-3′), IL-17A (FW 5′-CTG AAC ATC CAT AAC CGG AAT ACC A-3′, RV 5′-AGC GTT GAT GCA GCC CAA G-3′), RORγt (FW 5′-GCT GTG ATC TTG CCC AGA ACC-3′, RV 5′-TGC CCA TCA TCA TTG CTG TTA ATC C-3′), IL-22 (FW 5′-CCA GGC TCA GCA ACA GGC TAA-3′, RV 5′-TTT CAG CTT TGC TCT GGT CAA ATG-3′), IL-23 (FW 5′-CAG CTT TCA CAG AAG CTC TGC AC-3′, RV 5′-TGA CTG TTG TCC CTG AGT CCT TG-3′), IFN-γ (FW 5′-CTT AAA GAT GAC CAG AGC ATC CAA-3′, RV 5′-GGC GAC AGT TCA GCC ATC AC-3′), and IL-4 (FW 5′-AGC AGC TGA TCC GAT TCC TGA-3′, RV 5′-TCC AAC GTA CTC TGG TTG GCT TC-3′).

### CD4^+^ T Cell Purification and Stimulation

PBMCs were isolated from the blood samples freshly obtained from healthy controls and psoriasis patients by lymphocyte separation medium (PAA Laboratories GmbH, Austria) according to the manufacturer's instruction. For the positive selection of CD4^+^ T cells, MACS CD4 microbeads (Miltenyi Biotec, Auburn, CA, USA) were incubated with the PBMCs and applied to a MidiMACS separation column (Miltenyi Biotec, CA, USA). The purity of the CD4^+^ T cells was determined by flow cytometry to be >98% for each population. The purified CD4^+^ T cells were seeded onto U-bottom 96-well plates at 5 × 10^5^ cells/well in RPMI 1640 medium containing 10% fetal bovine serum with 1 μg/ml plate bound anti-CD3, 1 μg/ml plate bound anti-CD28, 30 U/ml rIl-2, and 10 ng/ml TGF-β with or without 50 ng/ml IL-21 for 3 days in all culture.

### CFSE Proliferation Assay

After isolation, CD4^+^ T cells were labeled using the CellTrace™ CFSE Cell Proliferation Kit (Invitrogen, CA, USA). In brief, the cells were incubated with 0.5 μM CFSE solution for 10 min on ice and then washed with and resuspended in cold RPMI 1640 medium containing 10% fetal bovine serum. After resuspension, the cells were seeded onto 96-well plates.

### Statistical Analysis

Statistical significance was calculated using SPSS 20.0. A significant difference analysis between the two groups was performed using two-tailed unpaired or paired Student's *t*-test. The correlation analysis was performed using the Pearson correlation test. For all statistical analyses, the data were considered significant when *P* < 0.05 (^*^), *P* < 0.01 (^**^), *P* < 0.001 (^***^) or *P* < 0.0001 (^****^).

## Results

### IL-21 and IL-21R Expression Are Increased in the Lesional Skin of Moderate-to-Severe Psoriasis Patients

**Figure 1:**
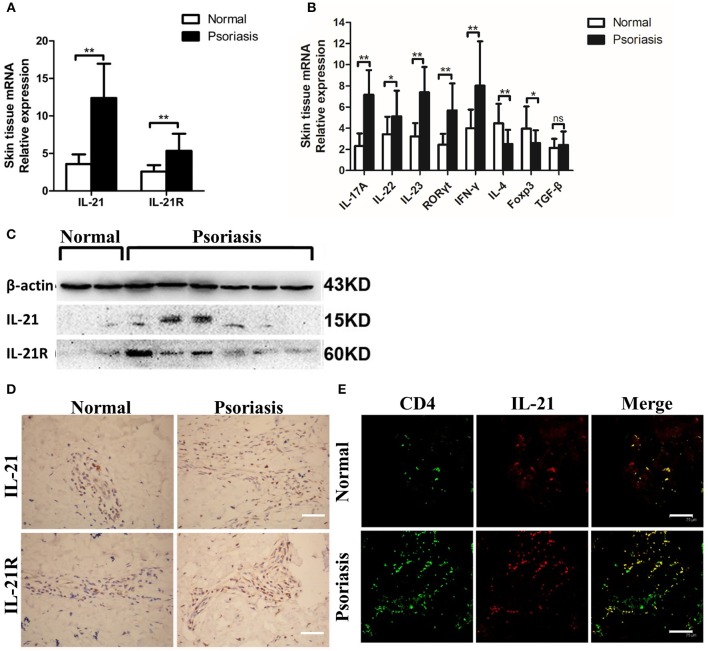
IL-21 and IL-21R are highly expressed in the lesional skin of psoriasis patients. **(A)** IL-21 and IL-21R mRNA expression in the skin of normal people (*n* = 15) and psoriasis patients (*n* = 25). **(B)** Expression levels of mRNA in the skin tissue of normal people (*n* = 15) and psoriasis patients (*n* = 25). **(C)** Western blotting of IL-21 and IL-21R of the skin of normal people (*n* = 15) and psoriasis patients (*n* = 25). **(D)** Immumohistochemical staining of IL-21 and IL-21R of the skin of normal people (*n* = 15) and psoriasis patients (*n* = 25). Bar = 75 μm. **(E)** Immunofluorescence staining of CD4 and IL-21 in skin paraffin sections obtained from normal people (*n* = 15) and psoriasis patients (*n* = 25). Bar = 75 μm. Data show means + SD. *P*-values were determined by unpaired Student's *t*-test. ^*^P < 0.05, ^**^P < 0.01.

First, we investigated whether IL-21 was increased in the psoriasis patients. Consistent with the previous studies (26–28), we found that the mRNA level of IL-21 and its receptor was higher in the lesional skin of psoriasis patients compared with the skin of healthy individuals (Figure 1A). The mRNA levels of IL-17A, IL-22, IL-23, RORγt, and IFN-γ were increased in the lesional skin of psoriasis patients (Figure 1B), whereas Foxp3, a vital transcriptional factor of Treg cells, was decreased in psoriasis patients (Figure 1B). The results of western blotting and immunohistochemical staining confirmed the higher expression of IL-21 and IL-21R in the protein level of psoriasis patients compared with the healthy individuals (Figures 1C,D). Furthermore, we found IL-21 was mainly expressed in CD4^+^ T cells in the skin tissues of both normal and psoriatic lesional skin as indicated in immunofluorescence staining (Figure 1E). Only a small percentage of IL-21 was expressed in other cell types, such as NK cells and B cells in psoriatic lesional skin (Supplementary Figure 1A). An increased expression of IL-17A and accumulation of Th17 cells were also found in the lesional skin of psoriasis patients (Supplementary Figures 2A,B). Overall, IL-21 and its receptor were highly expressed in CD4^+^ T cells in the lesional skin of moderate-to-severe plaque psoriasis patients.

### IL-21 and IL-21R Expression Are Increased in the PBMCs of Moderate-to-Severe Psoriasis Patients

**Figure 2:**
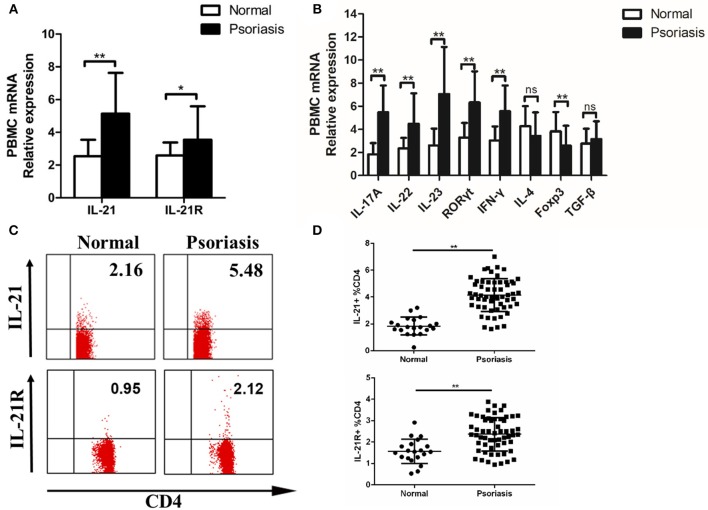
IL-21 and IL-21R expression are increased in the peripheral blood of psoriasis patients. **(A)** IL-21 and IL-21R mRNA expression in PBMCs of normal people (*n* = 20) and psoriasis patients (*n* = 58). **(B)** Expression levels of mRNA in PBMC are shown. Normal (*n* = 20), Psoriasis (*n* = 58). **(C–D)** Flow cytometry of IL-21 and IL-21R expression in CD4^+^ T cells from normal people (*n* = 20) and psoriasis patients (*n* = 58). Data show means + SD. *P*-values were determined by unpaired Student's *t*-test. ^*^P < 0.05, ^**^P < 0.01.

As psoriasis is a systemic disease, we tested the level of IL-21 in the peripheral blood of psoriasis patients and healthy individuals. Fresh PBMCs were isolated from the patients and healthy individuals. Then, we detected the expression of IL-21, IL-21R, and other cytokines or transcription factors. The mRNA levels of IL-21, IL-21R, IL-17A, IL-22, IL-23, RORγt, and IFN-γ were increased, whereas the mRNA level of Foxp3 was decreased in the PBMCs of psoriasis patients (Figures 2A,B). As IL-21 was mainly expressed in CD4^+^ T cells in the lesional skin of psoriasis patients, we then investigated the IL-21 expression in CD4^+^ T cells in PBMCs. Our result revealed that an increased number of CD4^+^ T cells secrete IL-21 and express IL-21R in the PBMCs derived from psoriasis patients (Figures 2C,D). In addition, we discovered that NK and B cells also expressed IL-21R (Supplementary Figure 1B). Overall, consistent with the findings of the lesional skin, IL-21 and its receptor were highly expressed in the CD4^+^ T cells of PBMCs of psoriasis patients.

### IL-21 Promotes CD4^+^ T Cell Proliferation and Th17 Cell Differentiation, Whereas Inhibits Treg Cell Differentiation

**Figure 3:**
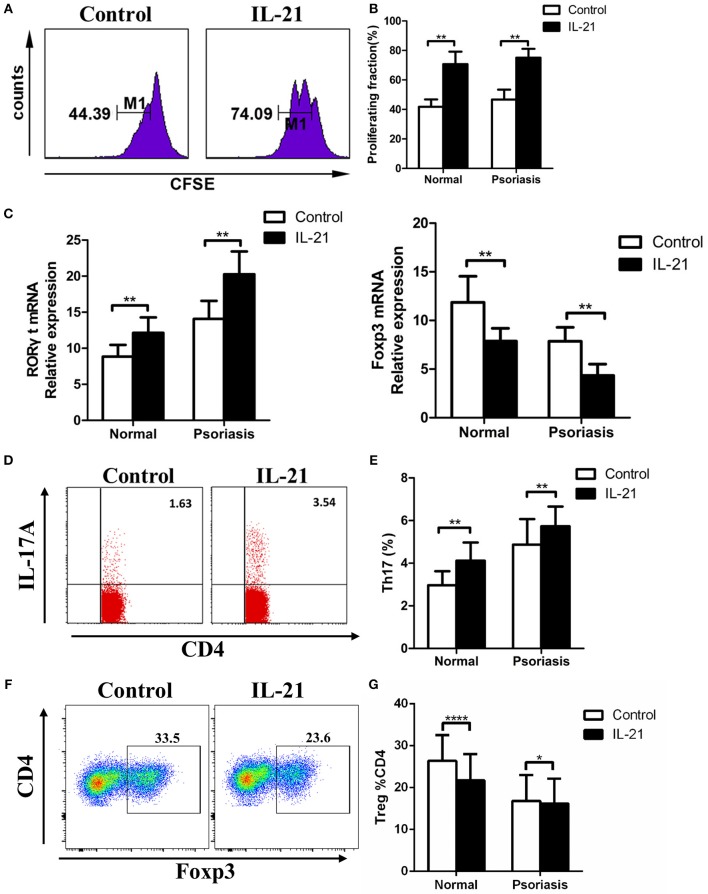
IL-21 promotes CD4^+^ T cell proliferation and Th17 cell differentiation, whereas inhibits Treg cell differentiation. **(A–B)** CFSE labeled CD4^+^ T cells from normal people (*n* = 8) and psoriasis patients (*n* = 12) were treated with or without IL-21 (50 ng/ml) for 3 days. **(A)** Showed a representative result of flow cytometry of psoriasis patients. **(C)** RORγt and Foxp3 mRNA expression of CD4^+^ T cells of normal people (*n* = 15) and psoriasis patients (*n* = 30) treated with or without IL-21 (50 ng/ml). **(D–E)** The proportion of Th17 cells (CD4^+^ IL-17A^+^ cells) in CD4^+^ T cells of normal people (*n* = 15) and psoriasis patients (*n* = 30) treated with or without IL-21 (50 ng/ml) for 3 days. **(D)** Showed a representative result of FACS of psoriasis patients. **(F–G)** The proportion of Treg cells (CD4^+^, CD25^+^, and Foxp3^+^ cells) in CD4^+^ T cells of normal people (*n* = 15) and psoriasis patients (*n* = 30) treated with or without IL-21 (50 ng/ml) for 3 days. **(F)** Showed a representative FACS result of normal people. Data show means + SD. *P*-values were determined by paired Student's *t*-test. ^*^P < 0.05, ^**^P < 0.01, ^****^P < 0.0001.

Previous studies have reported the multiple effects of IL-21 on CD4^+^ T cells in several diseases, so we hypothesized that IL-21 may also have effects on CD4^+^ T cells in psoriasis patients. CD4^+^ T cells were isolated from PBMCs derived from the psoriasis patients and healthy individuals, then treated with or without IL-21 for 3 days. After treatment, the proliferation rate and proportion of T cells subsets were determined using flow cytometry. Our results showed that more CD4^+^ T cells were proliferated after being stimulated with IL-21, both in psoriasis patients and healthy individuals (Figures 3A,B). The mRNA expression of RORγt, which plays a critical role in the differentiation of Th17 cells, was significantly increased after IL-21 stimulation (Figure 3C). We also found that the expression of Foxp3, which is essential for the differentiation and function of Treg cells, was reduced after the treatment with IL-21, both in psoriasis patients and healthy individuals (Figure 3C). Because of the up-regulation of RORγt and down-regulation of Foxp3, we speculated whether Th17 cell numbers were increased and Treg cell numbers were decreased after IL-21 treatment. We then tested the proportion of Th17 cells and Treg cells by using flow cytometry and found that the proportion of Th17 cells in the CD4^+^ T cells population was increased after the stimulation with IL-21 both in psoriasis patients and healthy individuals (Figures 3D,E), whereas the ratio of Treg cells was decreased after stimulation with IL-21 both in healthy individuals and psoriasis patients (Figures 3F,G). Overall, our data demonstrate that IL-21 can promote the proliferation of CD4^+^ T cells and the differentiation of Th17 cells and induce impaired Treg cell differentiation.

### IL-21 Increases Th17-Related Cytokines Expression and Regulates Treg-Related Cytokines Expression in CD4^+^ T Cells

**Figure 4:**
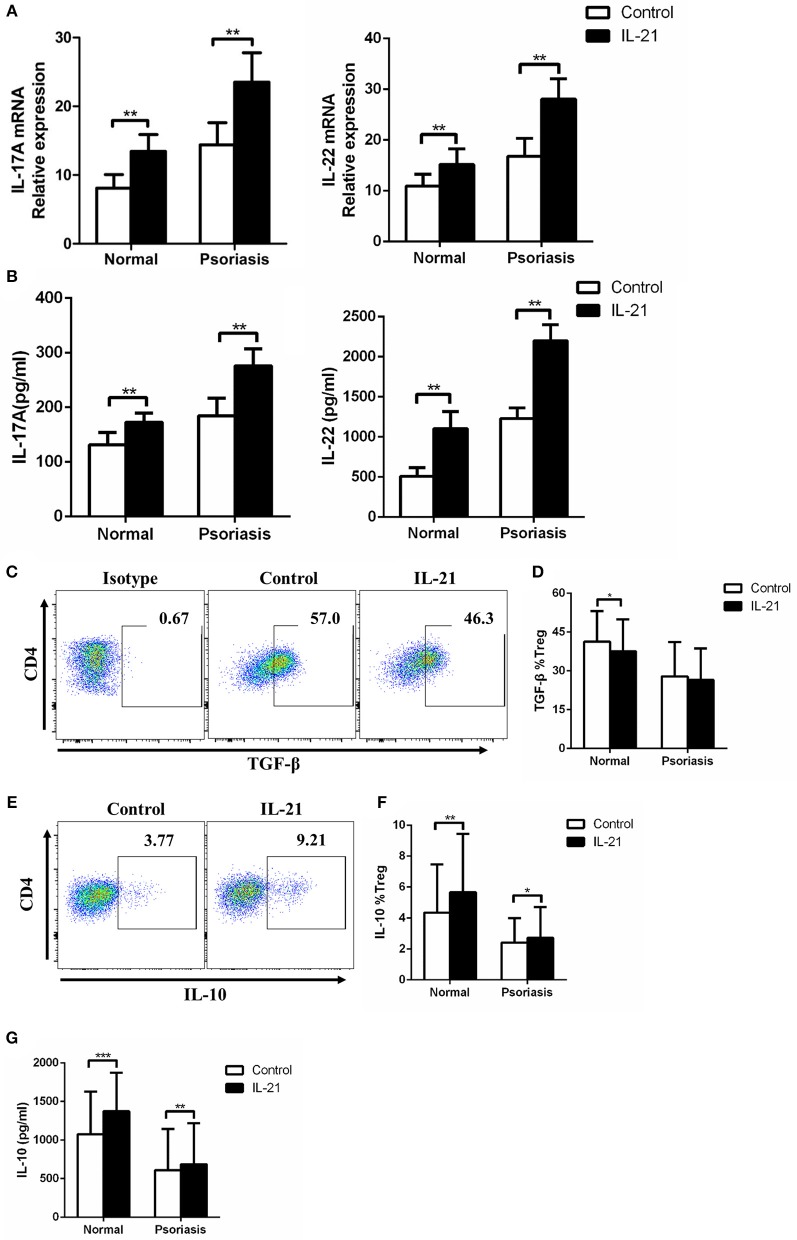
IL-21 increases Th17-related cytokines expression and regulates Treg-related cytokines expression in CD4^+^ T cells. **(A)** IL-17A and IL-22 mRNA expression of CD4^+^ T cells from normal people (*n* = 15) and psoriasis patients (*n* = 30) treated with or without IL-21 (50 ng/ml) for 3 days. **(B)** IL-17A and IL-22 were quantified in the supernatant of CD4^+^ T cells from normal people (*n* = 15) and psoriasis patients (*n* = 30) treated with or without IL-21 (50 ng/ml) for 3 days by ELISA. **(C–D)** The expression of TGF-β of Treg cells in CD4^+^ T cells of normal people (*n* = 15) and psoriasis patients (*n* = 30) treated with or without IL-21 (50 ng/ml) for 3 days. **(C)** Showed a representative FACS result of normal people. **(E–F)** The expression of IL-10 of Treg cells in CD4^+^ T cells of normal people (*n* = 15) and psoriasis patients (*n* = 30) treated with or without IL-21 (50 ng/ml) for 3 days. **(E)** Showed a representative FACS result of normal people. **(G)** IL-10 was quantified in the supernatant of CD4^+^ T cells from normal people (*n* = 15) and psoriasis patients (*n* = 30) treated with or without IL-21 (50 ng/ml) for 3 days by CBA. Data show means + SD. *P*-values were determined by paired Student's *t*-test. ^*^P < 0.05, ^**^P < 0.01, ^***^P < 0.001.

As IL-17A and IL-22 are the two key Th17-related cytokines that significantly contribute to the pathogenesis of psoriasis by inducing keratinocytes proliferation and pro-inflammatory cytokines production and secretion, we then investigated the expression of IL-17A and IL-22 in the CD4^+^ T cells treated with or without IL-21. We found that IL-17A and IL-22 mRNA expression and cytokines secretion were markedly increased after IL-21 stimulation (Figures 4A,B). Moreover, we examined Treg-related cytokines, including IL-10 and TGF-β. Our results showed that the expression of TGF-β was slightly decreased in Treg cells after stimulation with IL-21 in healthy individuals but not in psoriasis patients (Figures 4C,D). However, even though IL-21 is considered to be a pro-inflammatory cytokine, consistent with previous studies (29), we also found that IL-21 promoted the anti-inflammatory cytokine IL-10 expression in Treg cells (Figures 4E–G). These results indicate that the increased expression of IL-10 may be a compensatory mechanism for the reduction of Treg cells after IL-21 treatment. Overall, our results show that Th17-related cytokines expression was increased after IL-21 treatment.

### Imbalance of Th17 and Treg Cells Is Found in Psoriasis Patients

**Figure 5:**
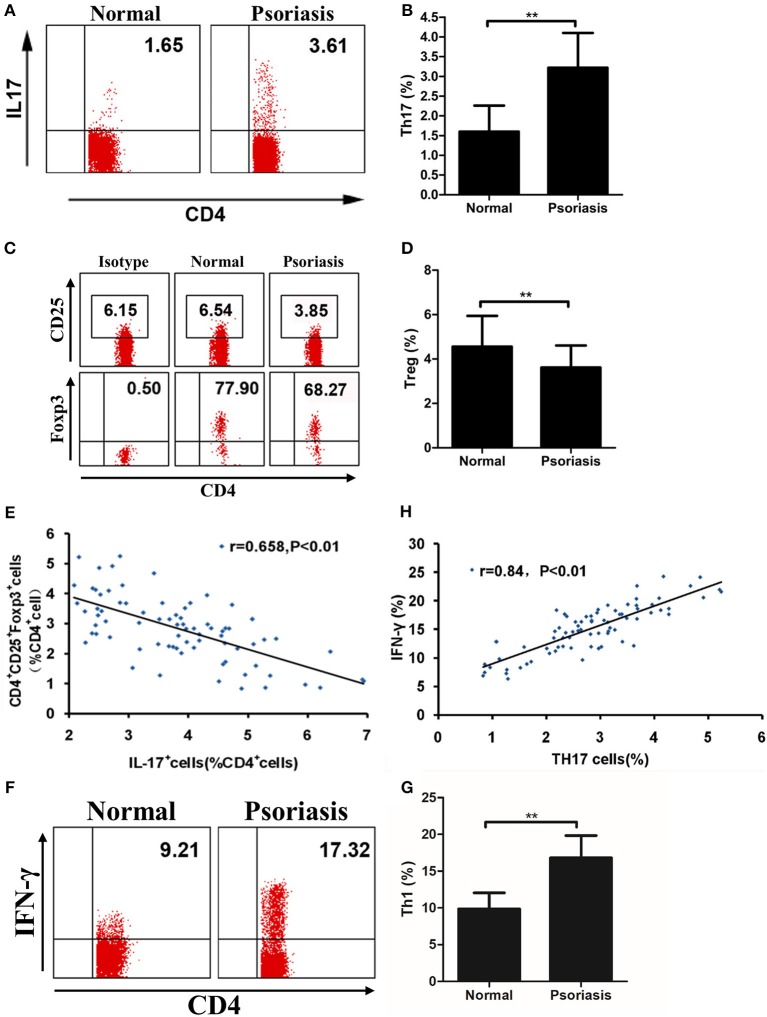
The proportion of Treg cells is negatively correlated with Th17 cells. **(A–B)** Proportion of Th17 cells in CD4^+^ cells of PBMCs from normal people (*n* = 20) and psoriasis patients (*n* = 58). **(C–D)** Proportion of CD25^+^Foxp3^+^ in CD4^+^ cells of PBMCs from normal people (*n* = 20) and psoriasis patients (*n* = 58). **(E)** Correlation between the proportion of Th17 cells and Treg cells of CD4^+^ cells of all participants (*n* = 78). **(F–G)** Proportion of Th1 cells in CD4^+^ cells of PBMCs from normal people (*n* = 20) and psoriasis patients (*n* = 58). **(H)** Correlation between the proportion of Th17 cells and Th1 cells of CD4^+^ cells of all participants (*n* = 78). Data show means + SD. *P*-values were determined by unpaired Student's *t-*test. ^**^P < 0.01.

As our data showed that IL-21 was highly expressed in psoriasis patients and promoted the proliferation of CD4^+^ T cells and the differentiation of Th17, we then investigated the proportion of Th17 and Treg cells in the PBMCs derived from psoriasis patients and healthy individuals. We found that the proportion of Th17 cells in CD4^+^ T cells was higher, whereas the proportion of Treg cells in CD4^+^ T cells was lower in psoriasis patients, compared with the healthy individuals (Figures 5A–D). We also found that the proportion of Treg cells was negatively correlated with that of Th17 cells (Figure 5E), while the proportion of Th1 cells (CD4^+^IFN-γ^+^) was increased in psoriasis patients (Figures 5F,G). Furthermore, the proportion of Th1 cells was positively correlated with that of Th17 cells (Figure 5H). Herein, the balance of Th17 and Treg cells is impaired in psoriasis patients, and this may be due to the upregulated expression of IL-21 in moderate-to-severe plaque psoriasis patients.

## Discussion

In the current study, IL-21 was significantly increased in both the lesional skin and peripheral blood of psoriasis patients consistent with the previous studies (26–28), suggesting the significant role of IL-21 in the pathogenesis of psoriasis. IL-21 is produced primarily by the CD4^+^ T cell population, with the highest production by T follicular helper cells and Th17 cells (24), and our data showed that IL-21 was mainly expressed in CD4^+^ T cells in the lesional skin.

Th17 cells play a critical role in the pathogenesis of psoriasis (5–7, 16, 30). Th17 cells are abundant in the lesional skin, and the proportion is markedly increased in the PBMCs of psoriasis patients (14, 31–33). Our data confirmed these findings. Th17 cells can promote the formation of psoriatic lesions by secreting certain inflammatory cytokines, including IL-17A and IL-22 (1, 34, 35). Several studies have shown that IL-21 induces T cell proliferation and promotes naïve Th0 cells to differentiate into Th17 cells (21, 22, 24, 36). Our results showed that IL-21R is highly expressed in PBMCs and in the lesional skin of psoriasis patients, and that IL-21 can promote CD4^+^ T cell proliferation and Th17 cell differentiation and also the secretion of IL-17A and IL-22. These results suggest that IL-21 participates in the pathogenesis of psoriasis via promoting T cell proliferation, inducing Th17 differentiation, and enhancing the function of Th17 cells.

In addition, Treg cells play a central role in maintaining immune homeostasis (19). Several studies have shown that the decreased number and/or impaired suppressive capacity of Treg cells are observed in autoimmune diseases (37–42). Treg cells can downregulate immune responses by expressing immunosuppressive proteins, such as CTLA-4 (42), or by secreting anti-inflammatory cytokines, such as IL-10 and TGF-β (43). Some studies have shown that the function of Treg cells is impaired in psoriasis patients, and Treg cells are easily converted into Th17 cells under psoriatic inflammatory conditions (18). Our data revealed that IL-21 could downregulate Foxp3 expression, which is a critical transcription factor in Treg cells, suggesting a role of IL-21 in the impaired function of Treg cells in psoriasis patients. As IL-21 expression was markedly increased in psoriasis patients and could promote CD4^+^ T cell proliferation and Th17 cell differentiation and also inhibited the expression of Foxp3, we then measured the proportion of Th17 and Treg cells in the PBMCs of psoriasis patients. We found that the proportion of Th17 cells was increased, whereas that of Treg cells was decreased, and these two proportions were negatively correlated. Hence, IL-21 plays a significant role in the imbalance of Th17 and Treg cells in psoriasis patients.

Several studies have shown that IL-21 plays a vital role in autoimmune diseases. IL-21 expression is significantly increased in some Th17-related autoimmune diseases, such as rheumatoid arthritis (44), multiple sclerosis (45), and inflammatory bowel disease (46, 47). IL-21 deficient mice showed decreased severity in these diseases models (21, 22, 48, 49) whereas in the imiquimod (IMQ)-induced psoriasis model, inflammation was not alleviated in the IL-21R deficient mice (50). As the main source of IL-17A in the psoriasis mouse model is γδT cells, not Th17 cells (11), and IL-21 can inhibit the secretion of IL-17A in γδT cells (51), no alleviation of inflammation can be observed in IL-21R deficient mice. Nevertheless, in the human psoriasis xenograft mouse model, the blockade of IL-21 alleviates skin inflammation, suggesting the critical role of IL-21 in human psoriasis (28).

**Figure 6:**
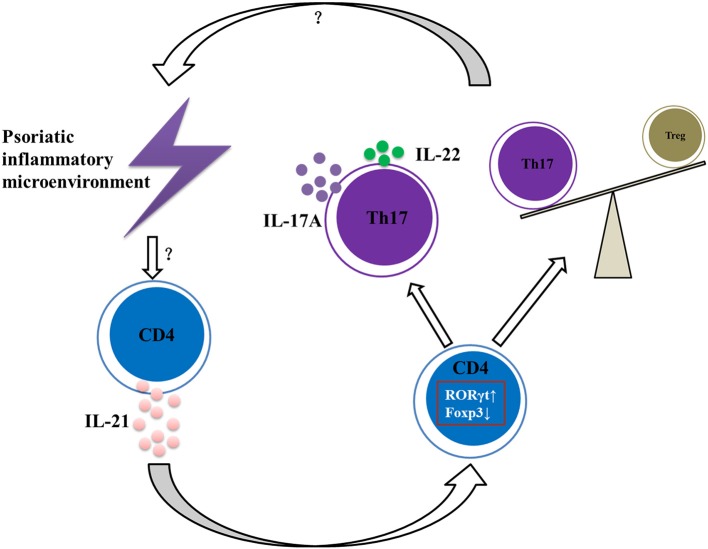
Summary of IL-21 actions in psoriatic inflammation. Under the psoriatic inflammatory microenvironment, CD4^+^ T cells secret abundant IL-21. Then IL-21 acts on CD4^+^ T cells via IL-21R to increase RORγt expression and decrease Foxp3 expression, resulting in imbalance of Th17 and Treg cells and increased expression of IL-17A and IL-22, further aggravating the inflammatory microenvironment of psoriasis.

In conclusion, our data suggest that IL-21 contributes to the pathogenesis of psoriasis by promoting CD4^+^ T cell proliferation, enhancing Th17 cell differentiation and function, downregulating the differentiation of Treg cells, and aggravating the imbalance of Th17 and Treg cells (Figure 6), and therefore IL-21 may be potential therapeutic targets in psoriasis.

## Data Availability

All datasets generated for this study are included in the manuscript and/or the Supplementary Files.

## Ethics Statement

This study was performed at the Shanghai Tenth People's Hospital and Shanghai Changhai Hospital and was approved by the ethics committees of those institutions.

## Author Contributions

YS, ZC, ZZ, YY, HF, XX, and XB conducted the experiments and analyzed the results. JG and YS planned the study and evaluated the results. ZC wrote the paper. YS edited the paper.

### Conflict of Interest Statement

The authors declare that the research was conducted in the absence of any commercial or financial relationships that could be construed as a potential conflict of interest.

## Supplementary Material

Supplementary Figure 1 A small percentage of IL-21 and IL-21R was expressed in NK cells and B cells. **(A)** Flow cytometry analysis of IL-21 expression in NK cells (CD16^+^ or CD56^+^) and B cells (CD19^+^) from lesional skin of psoriasis patients (*n* = 1). **(B)** Flow cytometry analysis of IL-21R expression in NK cells (CD16^+^ or CD56^+^) and B cells (CD19^+^) from PBMCs of psoriasis patients (*n* = 1).

Supplementary Figure 2 IL-17A is highly expressed in psoriatic lesional skin. **(A)** Immumohistochemical staining of IL-17A of the skin of normal people (*n* = 15) and psoriasis patients (*n* = 25). Bar = 75 μm. **(B)** Immunofluorescence staining of CD4 and IL-17A in skin paraffin sections obtained from normal people (*n* = 15) and psoriasis patients (*n* = 25). Bar = 75 μm.
