# Supplementary figures and images for: IL-21 Induces an Imbalance of Th17/Treg Cells in Moderate-to-Severe Plaque Psoriasis Patients
Source: Front Immunol. 2019 Aug 7;10:1865. doi: 10.3389/fimmu.2019.01865 (PMC6693306; doi:10.3389/fimmu.2019.01865)

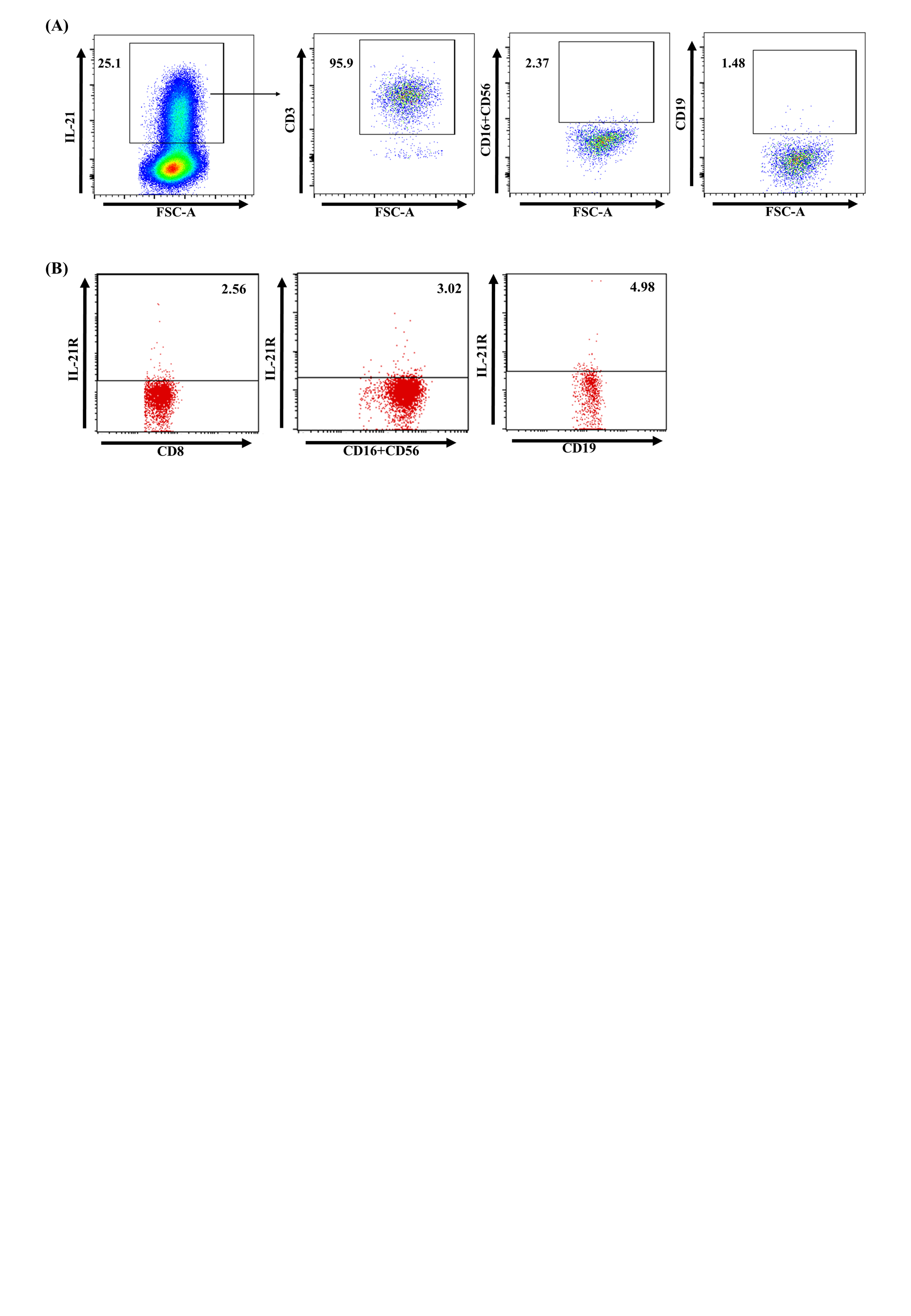

Supplement: Supplementary Figure 1 — A small percentage of IL-21 and IL-21R was expressed in NK cells and B cells. (A) Flow cytometry analysis of IL-21 expression in NK cells (CD16+ or CD56+) and B cells (CD19+) from lesional skin of psoriasis patients (n = 1). (B) Flow cytometry analysis of IL-21R expression in NK cells (CD16+ or CD56+) and B cells (CD19+) from PBMCs of psoriasis patients (n = 1). [file Image_1.TIF]

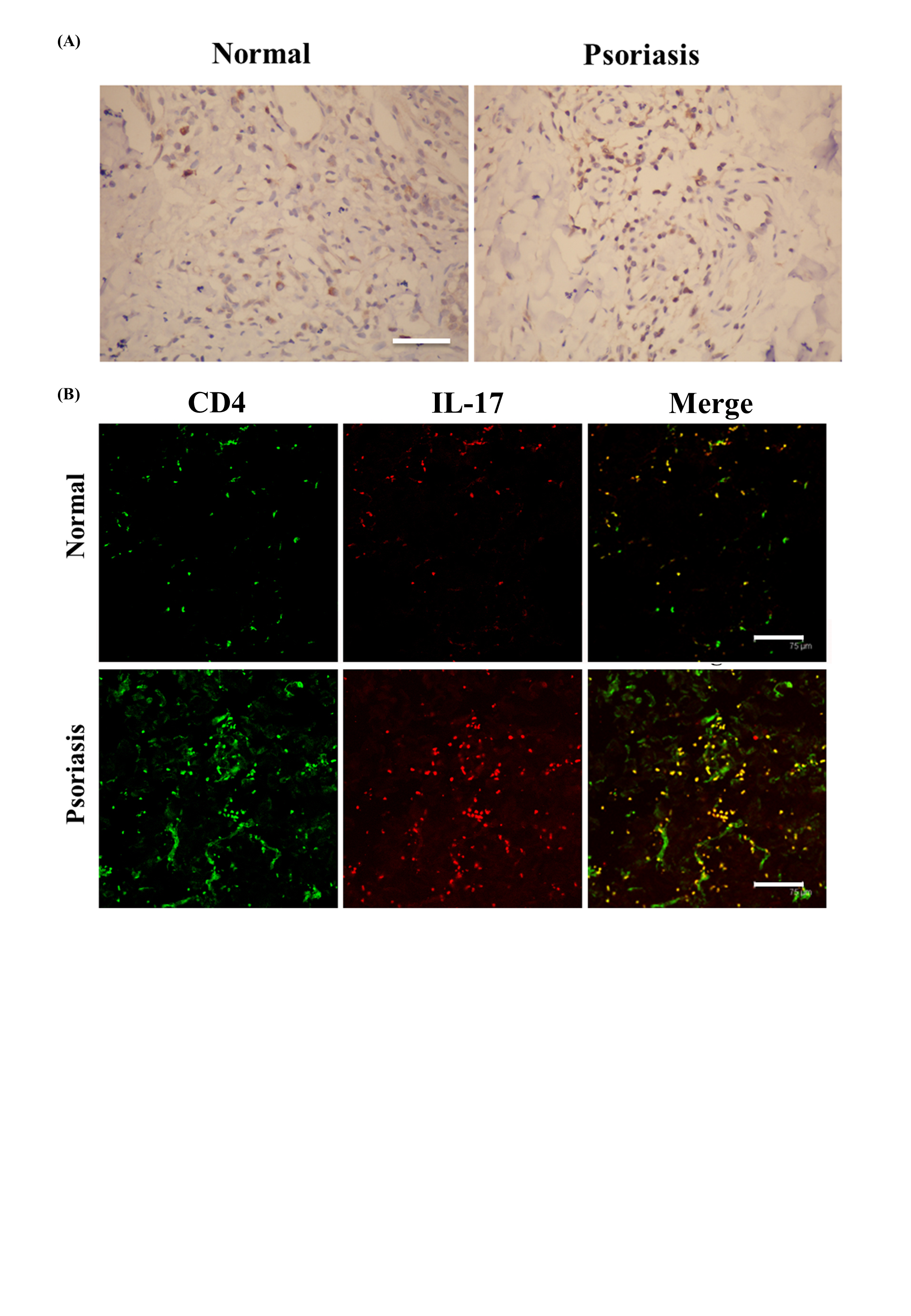

Supplement: Supplementary Figure 2 — IL-17A is highly expressed in psoriatic lesional skin. (A) Immumohistochemical staining of IL-17A of the skin of normal people (n = 15) and psoriasis patients (n = 25). Bar = 75 μm. (B) Immunofluorescence staining of CD4 and IL-17A in skin paraffin sections obtained from normal people (n = 15) and psoriasis patients (n = 25). Bar = 75 μm. [file Image_2.TIF]
